# Association of SARS-CoV-2 viral load distributions with individual demographics and suspected variant type: results from the Liverpool community testing pilot, England, 6 November 2020 to 8 September 2021

**DOI:** 10.2807/1560-7917.ES.2023.28.4.2200129

**Published:** 2023-01-26

**Authors:** David M Hughes, Christopher P Cheyne, Matthew Ashton, Emer Coffey, Alex Crozier, Malcolm G Semple, Iain Buchan, Marta García-Fiñana

**Affiliations:** 1 Department of Health Data Science, Institute of Population Health, University of Liverpool, Liverpool, United Kingdom; 2 Department of Public Health, Liverpool City Council, Liverpool, United Kingdom; 3 Division of biosciences, University College London, London, United Kingdom; 4 Health Protection Research Unity in Emerging and Zoonotic Infections, Institute of Infection, Veterinary and Ecological Science, University of Liverpool, United Kingdom; 5 Department of Public Health, Policy and Systems, Institute of Population Health, University of Liverpool, Liverpool, United Kingdom

**Keywords:** PCR cycle thresholds, COVID-19, age, variant, viral load

## Abstract

**Background:**

The PCR quantification cycle (C_q_) is a proxy measure of the viral load of a SARS-CoV-2-infected individual.

**Aim:**

To investigate if C_q_ values vary according to different population characteristics, in particular demographic ones, and within the COVID-19 pandemic context, notably the SARS-CoV-2 type/variant individuals get infected with.

**Methods:**

We considered all positive PCR results from Cheshire and Merseyside, England, between 6 November 2020 and 8 September 2021. C_q_ distributions were inspected with Kernel density estimates. Multivariable quantile regression models assessed associations between people’s features and C_q_.

**Results:**

We report C_q_ values for 188,821 SARS-CoV-2 positive individuals. Median C_q_s increased with decreasing age for suspected wild-type virus and Alpha variant infections, but less so, if not, for Delta. For example, compared to 30–39-year-olds (median age group), 5–11-year-olds exhibited 1.8 (95% CI: 1.5 to 2.1), 2.2 (95% CI: 1.8 to 2.6) and 0.8 (95% CI: 0.6 to 0.9) higher median C_q_s for suspected wild-type, Alpha and Delta positives, respectively, in multivariable analysis. 12–18-year-olds also had higher C_q_s for wild-type and Alpha positives, however, not for Delta. Overall, in univariable analysis, suspected Delta positives reported 2.8 lower median C_q_s than wild-type positives (95% CI: 2.7 to 2.8; p < 0.001). Suspected Alpha positives had 1.5 (95% CI: 1.4 to 1.5; p < 0.001) lower median C_q_s than wild type.

**Conclusions:**

Wild-type- or Alpha-infected school-aged children (5–11-year-olds) might transmit less than adults (> 18 years old), but have greater mixing exposures. Smaller differences in viral loads with age occurred in suspected Delta infections. Suspected-Alpha- or Delta-infections involved higher viral loads than wild type, suggesting increased transmission risk. COVID-19 control strategies should consider age and dominant variant.

**Key Public Health message**

**What did you want to address in this study?**

We questioned if people’s ability to transmit SARS-CoV-2 (infectiousness) varied with their age and the variant infecting them. As higher virus concentrations (viral loads) in respiratory samples have been reported to coincide with increased infectiousness, we quantified wild-type or variant SARS-CoV-2 in such samples, obtained from various age groups.

**What have we learnt from this study?**

Samples of individuals with suspected Delta variant had higher viral loads than samples with suspected wild-type or Alpha variant viruses. Hence, people with Delta variant may be more infectious. When wild-type and Alpha viruses circulated, secondary school aged children seemed to have lower viral loads than adults, but when Delta variant predominated this appeared not to be the case.

**What are the implications of your findings for public health?**

When attempting to control COVID-19 in an area, the SARS-CoV-2 variant that is affecting it should be taken in account, and measures should also consider demographic characteristics of inhabitants.

## Introduction

Testing has been an important tool among public health measures in controlling the spread of the severe acute respiratory syndrome coronavirus 2 (SARS-CoV-2) that causes coronavirus disease (COVID-19). Most countries have used reverse-transcription quantitative PCR (RT-qPCR) tests for clinical diagnosis of COVID-19 among those reporting symptoms. In the United Kingdom (UK), PCR tests are reported as binary positive/negative results.

Most laboratories also record qPCR results as the quantification cycle (C_q_) metric that reflects the concentration of viral genetic material in the sample [1]. Lower C_q_ values indicate higher viral concentrations, although the scale of this relation may vary between laboratories, making comparisons challenging [2-5]. In addition, the link between sample C_q_ and patient’s viral load is imprecise. Furthermore, individuals can show substantial differences in their viral shedding and replication patterns, therefore qPCR tests are not directly comparable with antigen tests [6].

Studies in the UK and Spain have shown that the viral load of an individual, approximated by qPCR C_q_ result, is associated with the risk of transmitting the virus to their contacts [7-9]. There is some evidence that C_q_ values may differ according to individuals’ features such as age, with young children having higher C_q_ values than adults [10-12]. Vaccination status and variant type may also influence C_q_ values [13,14].

From 6 November 2020, the City of Liverpool and the UK Department for Health and Social Care partnered in a pilot scheme to offer rapid SARS-CoV-2 antigen tests to people without symptoms of COVID-19, aiming to break chains of transmission by identifying a larger proportion of infected individuals, triggering more self-isolation [15]. Part of the pilot investigated the accuracy of lateral flow tests (LFTs) compared with qPCR [16]. During this pilot, a substantially increased number of qPCR as well as rapid SARS-CoV-2 antigen LFTs were conducted in Liverpool and its surrounding Cheshire and Merseyside region [17]. Additionally, at this time, the area was subject to various alert levels regarding COVID-19, according to a four-tier system [18], with restrictions on the population mixing increasing from Tier 1 to Tier 4.

In this paper, we describe the C_q_ distributions across various demographic features and variant types, for cases in Merseyside and Cheshire between 6 November 2020 and 8 September 2021. During the period in question, Liverpool experienced Tier 3 (early November 2020), national lockdown (November to early December 2020), Tier 2 (December 2020–early January 2021), national lockdown (January 2021–March 2021), the return to school (from early March 2021 onwards), and a period when the Delta variant was dominant in the UK (from mid-May 2021 onwards). Rates of vaccine coverage and variant types are reported elsewhere [19]. We investigate C_q_ distributions during these various policies of restrictions on mixing and by variant and individual characteristics.

## Methods

### Study design and participants

This study considers individual level, pseudonymised qPCR test results and demographic data provided by the Combined Intelligence for Population Health Action (CIPHA; www.cipha.nhs.uk) data resource. We have data on all Pillar 2 (individual-initiated community-based) qPCR tests conducted in Cheshire and Merseyside between 6 November 2020 and 8 September 2021.

Individuals were swabbed (combined nose and throat), and most swabs were sent to the Lighthouse Laboratories for qPCR testing, which uses Thermo Fisher PCR equipment in a standardised protocol, and their standard ThermoFisher TaqPath RT-qPCR SARS-CoV-2 assay. In the case where multiple positive qPCR tests were available per individual, we considered the first result that had C_q_ values available.

### Relationship between viral load and C_q_ values

As well as the binary qPCR test result, most positive test records also have three C_q_ values: for the spike protein encoding gene (S gene), the nucleocapsid encoding gene (N gene) and the open reading frame 1ab (ORF1ab), which encodes polyproteins. In this analysis we consider the mean of any gene C_q_ values present as representative of an individual’s sample C_q_.

We do not have a specific calibration curve for the laboratories in our study, however, Lighthouse Laboratories have performed a calibration on their protocol and equipment [8] which is considered here to be generalisable to our study. We report the proportion of individuals within demographic groups who have C_q_ < 18.3, 18.3– < 24.4, 24.4– < 30.5, ≥ 30.5 corresponding approximately to viral loads of > 1,000,000, > 10,000 –1,000,000, > 100 –10,000 and ≤ 100 RNA copies/mL respectively. This was obtained using a calibration curve for the Glasgow Lighthouse laboratory (log_10_(viral load) = 12 − 0.328 × C_q_, where C_q_ is the mean of any present of N-gene, S-gene and ORF1ab). The C_q_ thresholds chosen are for illustrative purposes only and are designed to allow comparability with previous work demonstrating the link between C_q_ and transmission of SARS-COV-2 [8].

### Demographic variables and individual characteristics

We consider the age, sex (male or female), and ethnicity (categorised as: white; black; asian; mixed or multiple ethnic groups; another ethnic group; or ’prefer not to say’) of the individual being tested, whether they were experiencing symptoms of COVID-19 at the time of test, whether the test was a home test kit or not, test administration method (self-administered, administered by a healthcare professional or unknown) and the time period in which the test was done. We also consider the number of LFTs taken by an individual in the 14 days before their positive test as an indicator of their engagement with asymptomatic testing (categorised as 0, 1 or ‘2 or more’). Since this was a ‘mass testing’ pilot, test seeking was not necessarily linked to the presence of symptoms, and asymptomatic testing was encouraged. We are not therefore able to assess time since symptom onset as a variable linked to C_q_ value.

We matched individuals to their vaccination record within CIPHA and include their vaccination status at the time of a positive test in the analysis, coded as 0, 1 or 2 doses.

### Time periods

We considered five periods; 6 November to 2 December 2020 (the initial ‘mass testing’ period during most of which there was a national lockdown), 3 December 2020 to 4 January 2021 (during which Liverpool was in Tier 2, with fewer restrictions than most of the country), 5 January to 7 March 2021 (during which the whole of England was back in a national lockdown and the Alpha variant was dominant), 8 March to 15 May 2021 (during which schools had reopened and secondary school children (aged 11–18 years) were encouraged to undertake lateral flow testing twice a week), and 16 May to 8 September (the period in which Delta variant was dominant, and restrictions were generally easing) [20]. It is possible that during these five periods, the differing levels of restrictions and changing immunity patterns (vaccine induced and naturally acquired) may have changed test seeking behaviours among individuals with COVID-19, in turn affecting observed viral loads. We included calendar time as a variable to see if there were noticeable differences in C_q_ approximated viral loads in different periods.

### Variant data

We do not have sequencing data available for the test results considered in this study. However, we used S gene target failure (absence of S gene amplification, but presence of N gene and ORF1ab) in RT-qPCR assays, as an indicator of likely SARS-CoV-2 ‘variant of concern’ (VOC) 122020/01 (Phylogenetic Assignment of Named Global Outbreak (Pango) lineage designation: B.1.1.7) named Alpha by the World Health Organization (WHO) [21]. This Alpha variant was first detected in the UK in November 2020. A further variant, initially detected in the UK in February 2021 and escalated to a VOC on 6 May (VOC-21APR-02; Pango lineage designation: B.1.617.2) was named Delta by the WHO on 31 May 2021. Unlike the Alpha variant, the Delta variant tests positive for S gene. Although not alone in this (wild-type, Beta, Kappa and other variants test positive for S gene), presence of S gene (and N gene and ORF1ab) in positive tests after 1 April 2021 is a useful proxy for presence of the Delta variant [22]. While the precise cut-off by 1 of April 2021 is debatable, fewer than 25% of sequenced positive qPCRs were wild type, falling to below 1% by the end of April. This makes 1 April 2021 a reasonable cut-off for determining likely Delta cases in the absence of sequencing data. 

In this study we therefore consider all three genes positive in tests before 1 April as likely original ‘wild-type’ SARS-COV-2, all three genes positive in tests from 1 April 2021 onwards as suspected Delta variant, all tests that are missing S gene but positive for N and ORF1ab as suspected Alpha variant, and all other tests as unknown variant status (e.g. only a single gene target positive, or only missing N or ORF1ab gene). We note that we use ‘wild-type SARS-CoV-2’ as this has become a common term but acknowledge that it was largely the D614G variant circulating in Europe since summer of 2020. No specific C_q_ value threshold was set for determining positivity [23].

### Statistical methods

The C_q_ value outcome was calculated as the mean of any C_q_ values for N gene, S gene or ORF1ab gene that were present. Kernel density estimates, with default oversmoothed bandwidth selection [24] were used to plot the distributions of C_q_ values across different demographic categories.

Since the mean C_q_ values were skewed left, we used median quantile regression to analyse the relationship between C_q_ values and demographic features. Firstly, each explanatory variable was considered in a univariate model to assess possible predictors of C_q_ independently. We then fit a multivariate quantile regression model, including all demographic variables considered in the univariate analyses, to allow adjustment for confounding between variables. As initial graphical explorations showed changes in the distributions of C_q_ per age group according to suspected variant type we included an interaction term between age group and suspected variant in the multivariate quantile regression model. Visual inspection of other variables by suspected variant did not show large differences, so we did not include further interaction terms for the sake of a more parsimonious model.

Statistical analysis was performed in R, version 3.6.1.

### Sensitivity analyses

We performed three additional sensitivity analyses. First, we restricted to cases where the mean C_q_ value was less than 31. This was to explore robustness of suspected variant designation by removing cases where S-gene dropout was caused by technical failure. Second, we considered cases where individuals had a positive LFT in the 14 days before the positive qPCR. Third, we considered the lowest C_q_ value per individual rather than the first positive qPCR in the case where multiple positive qPCRs were recorded for an individual.

## Results

Between 6 November 2020 and 8 September 2021, there were a total of 2,123,527 qPCR tests performed in the Cheshire and Merseyside region on 884,996 individuals, and 739,443 tests (35% of total tests) were on individuals who reported symptoms when booking the test. This yielded 208,315 positive results from 197,940 individuals (22% of individuals). C_q_ values were available for 188,821 individuals (95% of all positive cases).

Table 1 describes the numbers and results of tests for each group considered. The numbers of positive cases found over the whole study period, along with the median C_q_ values are shown in Supplementary Figure S1. There was a noticeable increase in the proportion of tests returning positive around the Christmas and New Year holiday period, and then again from July onwards as the Delta variant spread rapidly. However, the median levels of C_q_ values remained reasonably constant over the whole study period, although fluctuated more noticeably during April and May, while the prevalence of COVID-19 was low.

**Table 1:** Reverse-transcription quantitative PCR tests and their results according to demographic or individual characteristics, England, 6 November 2020‒8 September 2021 (n = 884,996 individuals)

Demographic or individual characteristics		Tests		Positive tests		Negative tests		Void tests		Number of people	Number with a positive test
		Number	% of total	Number	% of tests per category	Number	% of tests per category	Number	% of tests per category		
Total		2,123,527	100	208,315	10	1,887,710	89	27,502	1	884,996	197,940
Age group in years	5–11	85,738	4	12,345	14	72,145	84	1,248	1	59,200	11,926
	12–18	117111	6	23,990	20	91,378	78	1,743	1	75,211	22,879
	19–29	350,849	17	47,865	14	298,249	85	4,735	1	152,235	44,834
	30–39	358,196	17	37,477	10	316,334	88	4,385	1	144,364	35,521
	40–49	321,978	15	29,368	9	288,626	90	3,984	1	120,114	27,983
	50–59	385,390	18	28,302	7	352,324	91	4,764	1	125,395	27,001
	60–69	223,078	11	14,500	6	205,755	92	2,823	1	78,796	13,885
	≥ 70	187,375	9	9,090	5	175,796	94	2,489	1	72,975	8,703
	Missing age information^a^	93,812	3	5,378	6	87,103	93	1,331	1	56,706	5,208
Sex	Male	770,958	36	99,518	13	660,593	86	10,847	1	402,110	94,538
	Female	1,350,869	64	108,617	8	1,225,619	91	16,633	1	482,217	103,232
	Missing sex information^b^	1,700	0	180	11	1,498	88	22	1	669	170
Ethnicity	White	1,843,612	87	190,306	10	1,629,470	88	23,836	1	797,306	181,117
	Black	24,984	1	1,903	8	22,726	91	355	1	8,059	1,769
	Asian	40,936	2	4,550	11	35,768	87	618	2	19,174	4,225
	Mixed/multiple ethnic groups	24,202	1	3,018	12	20,842	86	342	1	12,824	2,861
	Another ethnic group	10,076	0	1,501	15	8,379	83	196	2	5,409	1,375
	Prefer not to say	179,717	8	7,037	4	170,525	95	2,155	1	42,224	6,593
Symptoms reported	Yes	739,443	35	161,078	22	567,542	77	10,823	1	480,305	154,069
	No	1,384,084	65	47,237	3	1,320,168	95	16,679	1	404,305	43,871
Home test	Yes	367,377	17	40,410	11	320,247	87	6,720	2	182,633	38,007
	No	1,756,150	83	167,905	10	1,567,463	89	20,782	1	702,363	159,933
Administration method	Healthcare professional	141,193	7	9,634	7	130,044	92	1,515	1	50,184	9,208
	Self-administered	1,353,140	64	163,168	12	1,172,777	87	17,195	1	618,397	155,487
	Not known	629,194	30	35,513	6	584,889	93	8,792	1	216,415	33,245
Test period	6 Nov–2 Dec	176,741	8	12,188	7	162,117	92	2,436	1	109,343	11,652
	3 Dec–4 Jan	203,698	10	31,175	15	169,504	83	3,019	1	117,765	29,243
	5 Jan–7 Mar	485,927	23	58,711	12	419,521	86	7,695	2	202,009	55,188
	8 Mar–16 May	346,117	16	4,351	1	338,502	98	3,264	1	85,783	4,228
	17 May–8 Sep	911,044	43	101,890	11	798,066	88	11,088	1	370,096	97,629
Variant	Unknown^c^/negative	1,925,435	91	12,149	1	1,886,807	98	26,479	1	743,181	11,372
	Wild type	35,732	2	35,511	99	0	0	221	1	26,912	33,431
	Suspected Alpha	62,535	3	61,910	99	0	0	625	1	45,238	58,350
	Suspected Delta	99,825	5	98,745	99	903	1	177	0	69,665	94,787
Vaccine doses	0	1,098,368	52	152,078	14	930,059	85	16,231	1	599,211	143,945
	1	353,409	17	22,788	6	326,354	92	4,267	1	100,912	21,787
	2	671,750	32	33,449	5	631,297	94	7,004	1	184,872	32,208

N gene: nucleocapsid gene; ORF1ab: open reading frame 1ab; RT-qPCR: reverse-transcription quantitative PCR.

^a^ Pillar 2 testing was available to over 5 year olds. Any age recorded of 4 years or less was assumed to be a data entry error and is recorded as missing in this table.

^b^ A small number of tests were missing data on the individuals sex.

^c^ Samples showing only a single gene target positive by RT-qPCR, or only missing N or ORF1ab gene were classified as having unknown variant status.

### Age

**Figure 1:**
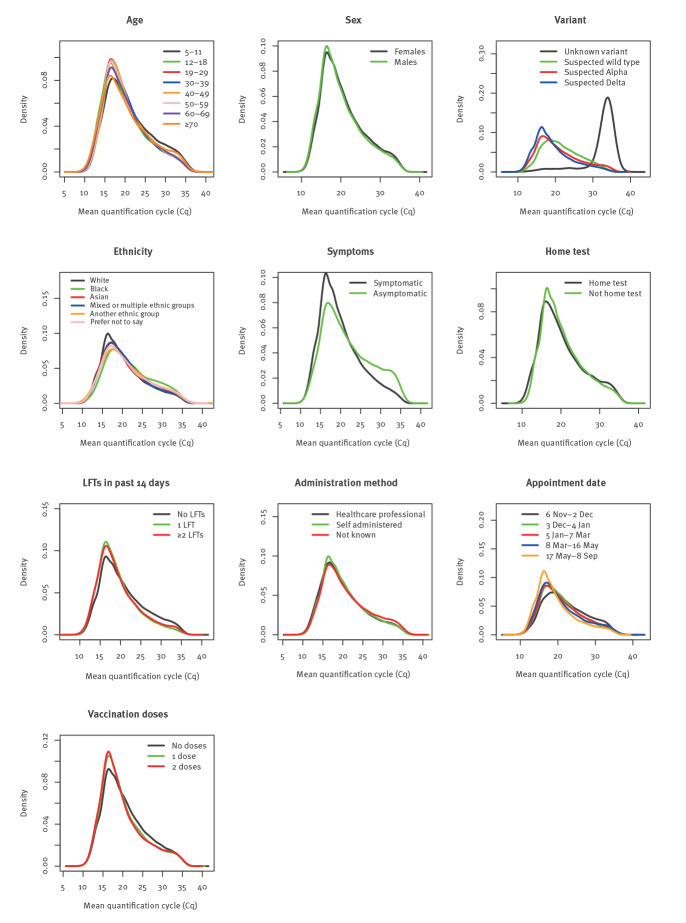
Distribution of RT-qPCR quantification cycle (C_q_) values according to different individual characteristics, England, 6 November 2020‒8 September 2021 (n = 188,821 individuals)

The distribution of age within our cohort is shown in Supplementary Figure S2. The median age of the positive cases was 33 years (inter-quartile range (IQR): 21–49). The youngest age group (5–11-year-olds) showed higher median C_q_ values (19.8; IQR: 16.5–24.7) than the rest of the population (19.0; IQR: 16.2–23.4), with higher proportions of positive tests showing C_q_ values in the > 30.5 range (8% of 5–11-year-olds compared to 6% of over 11’s, Supplementary Table S1). The distributions of mean C_q_s for most age categories were similar, although the distributions for 5–11-year-olds (primary school children) were noticeably skewed towards higher C_q_s (Figure 1).

RT-qPCR: reverse-transcription quantitative PCR.

The y-axis shows a kernel density estimate of the probability of observing each C_q_ value.

**Figure 2:**
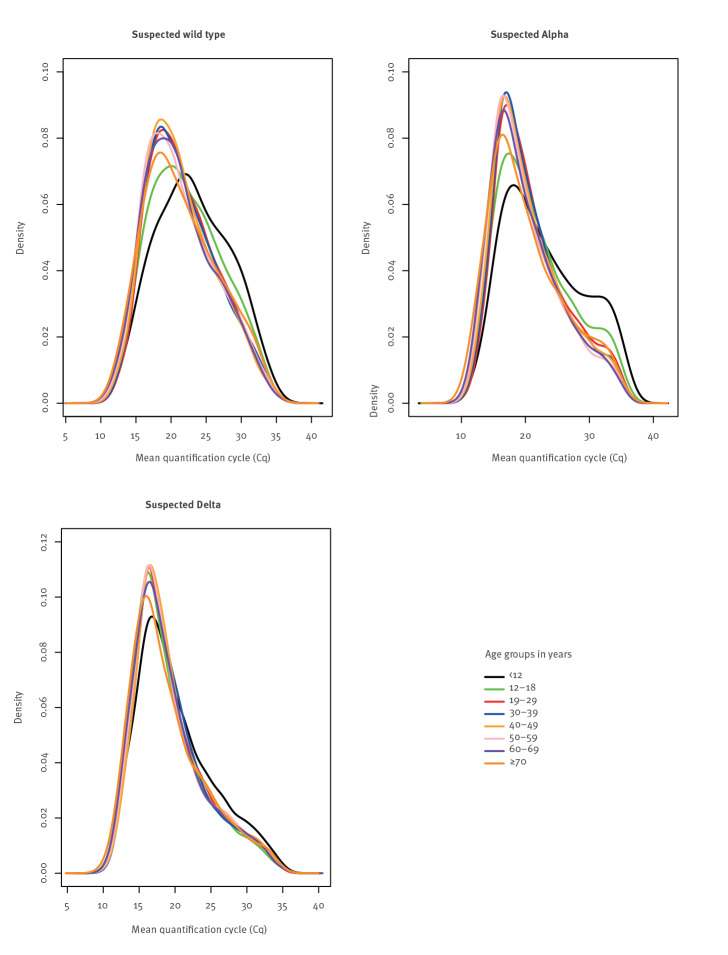
Distribution of RT-qPCR quantification cycle (C_q_) values according to suspected variant type and by age group, England, 6 November 2020‒8 September 2021 (n = 184,899 individuals)^a^

Several previous studies reported higher C_q_ values in 12–18-year-olds than in adult populations (aged > 18 years old), but this was not apparent in Figure 1 [10-12]. However, when broken down by suspected variant, 12–18-year-olds do appear to have C_q_ distributions that are more right skewed than most adult age groups (> 18 years old), along with the 5–11-year-olds, for both suspected wild-type SARS-CoV-2 and suspected Alpha variant (Figure 2), but not for Delta variant.

Cq: quantification cycle; RT-qPCR: reverse-transcription quantitative PCR.

^a ^As only individuals with data on age are included, the total is different from 188,821.

The y-axis shows a kernel density estimate of the probability of observing each mean C_q_ value.

Quantile regression models estimated that 5–11-year-olds had positive qPCRs with C_q_ values 1.81 units higher (95% confidence interval (CI): 1.47 to 2.10) than 30–39-year-olds (who contained the median age) for suspected wild-type positives (Table 2), 2.20 units higher for suspected Alpha positives (95% CI: 1.81 to 2.58) and 0.75 (95% CI: 0.58 to 0.91) units higher for suspected Delta positives (Supplementary Table S2). Those 12–18-years-old also showed higher C_q_ values than 30–39-year-olds during wild-type (Table 2) and Alpha (Supplementary Table S2) waves with C_q_ values 0.81 (95% CI: 0.51 to 1.07) and 0.81 (95% CI: 0.55 to 1.07) units higher respectively. However, this increase was not observed for suspected Delta positives, with in fact a 0.18 unit reduction in C_q_ (95% CI: 0.06 to 0.30) relative to 30–39-year-olds. Other age groups also showed statistically significant differences in C_q_ distribution relative to 30–39-year-olds, but the magnitude of the difference was generally smaller than was observed for 5–11-, and 12–18-year-olds, except for over-70-year-olds with suspected Alpha or Delta variants (Supplementary Table S2).

**Table 2:** Quantile (median) regression model results showing the association between demographic or individual features and RT-qPCR quantification cycle values, England, 6 November 2020‒8 September 2021 (n = 184,899 individuals)^a^

Demographic or individual features		Univariate models			Multivariate model		
		Parameter	95% CI	p value	Parameter	95% CI	p value
Age groups in years^b^	30–39	Reference					
	5–11	0.77	0.64 to 0.92	< 0.001	1.81	1.47 to 2.10	< 0.001
	12–18	−0.55	−0.64 to −0.45	< 0.001	0.81	0.51 to 1.07	< 0.001
	19–29	−0.10	−0.18 to −0.02	0.037	0.16	−0.03 to 0.34	0.159
	40–49	−0.02	−0.12 to 0.08	0.713	−0.21	−0.41 to −0.01	0.066
	50–59	−0.17	−0.27 to −0.07	0.002	−0.33	−0.53 to −0.16	0.005
	60–69	−0.11	−0.23 to −0.01	0.11	−0.33	−0.56 to −0.12	0.017
	≥ 70	−0.03	−0.18 to 0.13	0.773	−0.25	−0.56 to 0.02	0.160
Sex	Female	Reference					
	Male	−0.47	−0.51 to −0.42	< 0.001	−0.37	−0.41 to −0.33	< 0.001
Symptomatic	No	Reference					
	Yes	−1.71	−1.79 to −1.64	< 0.001	−1.65	−1.72 to −1.58	< 0.001
Ethnicity	White	Reference					
	Prefer not to say	0.79	0.63 to 0.98	< 0.001	0.44	0.31 to 0.56	< 0.001
	Black	1.77	1.41 to 2.15	< 0.001	1.44	1.13 to 1.77	< 0.001
	Asian	0.45	0.26 to 0.65	< 0.001	0.17	−0.02 to 0.32	0.080
	Another ethnic group	0.80	0.58 to 1.17	< 0.001	0.55	0.30 to 0.83	0.001
	Mixed or multiple ethnic groups	0.44	0.25 to 0.63	< 0.001	0.57	0.38 to 0.78	< 0.001
Home test	No	Reference					
	Yes	−0.12	−0.18 to −0.05	0.003	−0.12	−0.18 to −0.06	< 0.001
Possible variant	Wild type	Reference					
	Alpha	−1.45	−1.53 to −1.38	< 0.001	−1.62	−1.81 to −1.45	< 0.001
	Delta	−2.79	−2.85 to −2.72	< 0.001	−7.51	−8.31 to −6.92	< 0.001
	Unknown	12.67	12.55 to 12.74	< 0.001	10.86	10.55 to 11.23	< 0.001
Test period	6 Nov–2 Dec	Reference					
	3 Dec–4 Jan	−1.12	−1.27 to −0.98	< 0.001	−0.15	−0.29 to −0.03	0.037
	5 Jan–7 Mar	−1.15	−1.29 to −1.03	< 0.001	0.45	0.32 to 0.59	< 0.001
	8 Mar–16 May	−1.84	−2.03 to −1.60	< 0.001	0.34	0.14 to 0.56	0.023
	17 May–8 Sep	−2.85	−2.98 to −2.74	< 0.001	4.72	4.12 to 5.57	< 0.001
Administration method	Healthcare professional	Reference					
	Unknown	0.49	0.34 to 0.64	< 0.001	0.24	0.12 to 0.39	0.003
	Self-administered	0.07	−0.06 to 0.20	0.429	0.18	0.08 to 0.33	0.011
LFTs in previous 14 days before positive PCR	0	Reference					
	1	−1.21	−1.27 to −1.15	< 0.001	−1.06	−1.11 to −1.00	< 0.001
	≥ 2	−1.11	−1.19 to −1.04	< 0.001	−1.00	−1.06 to −0.92	< 0.001
Number of vaccine doses	0	Reference					
	1	−0.83	−0.89 to −0.75	< 0.001	0.34	0.28 to 0.42	< 0.001
	2	−0.95	−1.01 to −0.89	< 0.001	0.56	0.47 to 0.65	< 0.001
**Interactions between virus variant and age group in years**							
Alpha * 5–11	NA	NA	NA	NA	0.39	−0.08 to 0.88	0.155
Alpha * 12–18	NA	NA	NA	NA	0	−0.38 to 0.35	0.989
Alpha * 19–29	NA	NA	NA	NA	0.15	−0.06 to 0.4	0.292
Alpha * 40–49	NA	NA	NA	NA	0.08	−0.17 to 0.33	0.596
Alpha * 50–59	NA	NA	NA	NA	−0.22	−0.45 to 0.03	0.128
Alpha * 60–69	NA	NA	NA	NA	−0.23	−0.53 to 0.05	0.186
Alpha * 70 +	NA	NA	NA	NA	−0.55	−0.89 to −0.17	0.017
Delta * 5–11	NA	NA	NA	NA	−1.06	−1.42 to −0.69	< 0.001
Delta * 12–18	NA	NA	NA	NA	−0.99	−1.27 to −0.67	< 0.001
Delta * 19–29	NA	NA	NA	NA	−0.1	−0.31 to 0.1	0.419
Delta * 40–49	NA	NA	NA	NA	0.09	−0.13 to 0.33	0.491
Delta * 50–59	NA	NA	NA	NA	−0.04	−0.24 to 0.21	0.778
Delta * 60–69	NA	NA	NA	NA	−0.13	−0.4 to 0.15	0.411
Delta * ≥ 70	NA	NA	NA	NA	−0.44	−0.77 to −0.09	0.037
Unknown * 5–11	NA	NA	NA	NA	−2.88	−3.44 to −1.8	< 0.001
Unknown * 12–18	NA	NA	NA	NA	−1.95	−2.91 to −1.31	< 0.001
Unknown * 19–29	NA	NA	NA	NA	−1.12	−1.9 to −0.53	< 0.001
Unknown * 40–49	NA	NA	NA	NA	−0.25	−0.72 to 0.21	0.084
Unknown * 50–59	NA	NA	NA	NA	0.05	−0.42 to 0.54	0.839
Unknown * 60–69	NA	NA	NA	NA	0.68	0.15 to 1.31	0.002
Unknown * ≥ 70	NA	NA	NA	NA	0.22	−0.21 to 0.64	0.286

CI: confidence interval; LFT: lateral flow test; NA: not applicable; RT-qPCR: reverse-transcription quantitative PCR.

^a ^Individuals with missing age or sex information were excluded, hence the total here is different from 188,821.

^b^ This is interpreted as the age effect for people with wild-type virus suspected infection only, with the same comparison for people with Alpha or Delta shown separately in Supplemental Table S2.

### Sex and ethnicity

Median regression models revealed that males had a C_q_ median value which was lower than females by 0.37 (95% CI: 0.33 to  0.41; p < 0.001). For ethnicity, most comparisons (with white individuals as the reference group) showed statistical significance, but with small differences in median C_q_ (after adjustment for other variables). Most noticeably, individuals of black ethnicity showed higher C_q_ values than white ethnic groups (1.44 C_q_ values higher; 95% CI: 1.13 to 1.77, Figure 1 and Table 2).

### Symptom status

Individuals who did not report symptoms when tested had generally higher C_q_ values, with 31% (12,880/41,222) of non-symptomatic individuals having C_q_ > 24.4 compared with 19% (27,910/147,599) in individuals who reported symptoms (Supplementary Table S1). Symptomatic individuals had C_q_ 1.65 units lower (95% CI:  1.58 to 1.72) than asymptomatic individuals in the multivariable model, which adjusted for other features (Table 2).

### Variant

Figure 1 suggests a difference in the distribution of the C_q_ values based on variant type, with positive tests suspected to be either Alpha or Delta variants reporting lower C_q_ values (higher viral loads). Multivariable regression shows tests with the possible Alpha variant had C_q_ values 1.62 (95% CI: 1.45 to 1.81) and suspected Delta variant 7.51 (95% CI: 6.92 to 8.31) units lower than the wild-type SARS-CoV-2 (note that the effect size was smaller, though still noteworthy in univariate models). Tests with unknown variant type (single gene positives etc.) reported much higher C_q_ values.

**Figure 3:**
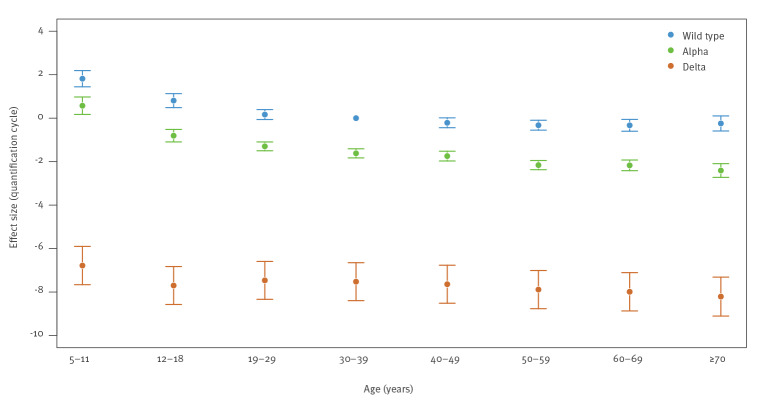
Association between median RT-qPCR quantification cycle^a^, and age, variant interactions, England, 6 November 2020‒8 September 2021 (n = 184,899 individuals)^a^

Figure 3 shows the interaction between age and suspected variant type. This confirms that suspected Delta cases had much lower C_q_ values than wild-type and shows the reduction in age effect in Delta cases. For suspected Alpha and wild-type cases the higher C_q_ values for 5–11-, and 12–18-year-olds are noticeable.

RT-qPCR: reverse-transcription quantitative PCR.

^a ^Individuals with missing age or sex information were excluded, hence the total here is different from 188,821.

The point estimates represent the association between age and median quantification cycle from the quantile regression model, while the intervals are 95% confidence intervals around these effect sizes. Contrasts are relative to the 30–39-year-old group with wild-type severe acute respiratory coronavirus 2.

**Figure 4:**
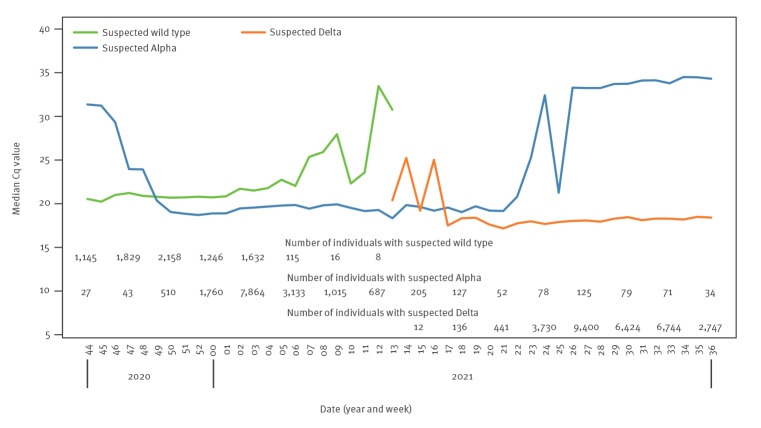
Median of mean RT-qPCR quantification cycle values over time for each variant type, England, 6 November 2020‒8 September 2021 (n = 188,821 individuals)

Figure 4 shows the changes over time in the median of mean C_q_ values by week, for each suspected variant type. In general, this supports the conclusion that the Alpha variant cases have on average a lower C_q_ (higher viral load) than wild-type SARS-COV-2 and that Delta variant cases have even higher viral loads on average. The high C_q_ for suspected Alpha variant before week 48 of 2020 (week beginning 29 November 2020) likely reflects a high proportion of positive tests where the missing S gene was a technical failure rather than an indication of the Alpha variant (i.e. wild-type cases where a gene was not detected). This may also be true for the observed increase after week 21 of 2021.

Cq: quantification cycle; N gene: nucleocapsid gene; ORF1ab: open reading frame 1ab; RT-qPCR: reverse-transcription quantitative PCR; S gene: spike protein encoding gene.

Suspected Alpha is defined as a positive sample with missing S gene but present N and ORF1ab gene targets. Suspected Delta was defined as positive tests having all three target genes present and the test occurring after 1 April 2021. Positive tests with all three target genes present before 1 April 2021 were defined as suspected ‘wild type’. The number of individuals with each variant type is shown for every third week for the sake of clarity.

### Appointment date

Differences in C_q_ distributions were observed for the five time periods considered in this analysis (Table 2), with C_q_ values decreasing as time progressed from November onwards (Figure 1). Although statistically significant, most of the effect sizes decrease in the multivariate model compared with the univariate model (Table 2), explained by the high correlation between the study periods and the suspected variant.

### Number of lateral flow tests in the 14 days before a positive test

Of 184,899 individuals with information on all variables, approximately 75.6% (n = 139,839) had taken no LFTs in the 14 days before their positive qPCR, with 17.3% (n = 32,050) taking one LFT and 7.1% (n = 13,010) taking two or more. Individuals with at least one LFT in the 14 days before their positive qPCR reported a 1-unit lower C_q_ values than individuals who had no LFTs during the period. A total of 28,251 individuals had a positive LFT in the 14 days before a positive qPCR, corresponding to 15.3% of all individuals and 62.7% of individuals with at least one LFT (n = 45,060).

### Vaccination status

In the adjusted quantile regression model, individuals with more vaccine doses were associated with higher C_q_ values (Table 2), although the effect sizes are relatively small, and Figure 1 does not show substantial difference in C_q_s dependent on number of vaccine doses. Further, for the Delta variant, Supplementary Figure S7 does not reveal clear differences in C_q_ values for different age groups across vaccination status of individuals at the time of a positive test.

Minor differences were observed for sex, test location and administration method with further details reported in the supplementary material.

### Sensitivity analyses

Sensitivity analyses showed that, compared to wild-type or Alpha cases, the lower C_q_ values for suspected Delta cases, and, compared to 30–39-year-olds, the higher C_q_ values for 5–11- and 12–18-year-olds with suspected wild-type or Alpha infections, but only for 5–11 year olds with Delta infections, remained evident when considering (i) only individuals with mean C_q_ < 31 (Supplementary Figures S11 and S12 and Table S5), (ii) individuals with a positive LFT in the 14 days before the positive qPCR (Supplementary Figures S13 and S14 and Table S5) and (iii) the lowest C_q_ value per individual (Supplementary Figures S15 and S16 and Table S5). This reinforces the findings of our analysis.

## Discussion

This large study investigated the influence of demographic and individual features on qPCR C_q_ values. While all features demonstrated statistically significant differences in median C_q_, in most cases visual inspection of the distributions did not reveal large, clinically relevant differences, suggesting the statistically significant differences were a result of the very large sample size.

Our finding that males had C_q_ values ca 0.37 units lower than females is consistent with the finding of an Office for National Statistics (ONS) survey published in 2021, that reported 0.3 units lower (95% CI: 0.1 to  0.5; p = 0.001) [23]. We observed higher C_q_ for non-white ethnicities whereas the ONS study detects lower C_q_ values in non-white individuals, though these differences are insignificant on adjusted analyses [23]. The ONS survey uses random sampling and could be detecting individuals at a later stage than those attending test centres in our cohort. There may be differences in the time at which different ethnic groups seek a test in our cohort, or the likelihood of responding to surveys. This apparent discrepancy may be worth further investigation, although the differences in C_q_ for different ethnicities were generally small except for people of black ethnicity.

School-aged children generally had higher C_q_ values. However, this was not the case for suspected cases of Delta variant, where only 5–11-year-olds showed higher C_q_ values and these were just slightly higher. A Dutch study, published in 2021, performed before Delta variant was widespread, showed that children aged under 12 and 12–18 years have lower viral loads than older individuals irrespective of sex and symptom duration [10]. Their study considered symptomatic individuals, mainly in hospital and general practitioner (GP) based test settings. Our study confirms their findings in a community testing setting with largely asymptomatic participants, during the period in which wild type or Alpha were prominent (Figure 3).

A study in Switzerland did not reveal significant differences in C_q_ across age groups, although it only included 77 individuals aged under 19 years [11]. Similarly, a study in Germany reported no difference between age groups [12]. However, subsequent statistical critique of the German study suggested a moderate age effect on reanalysis [25]. A 2021 ONS survey reported no significant association between age and C_q_ value [23]. However, examination of their supplementary material shows that primary school aged children were associated with 1 C_q_ higher (95% CI: 0.3 to 1.7) than 17–24-year-olds which is consistent with our findings in Supplementary Table S2. Most of the age associations reported in their supplement appear consistent with ours (allowing for different reference groups). Both this and our study demonstrate that while differences in C_q_ between different age groups are generally small, school aged children, and particularly primary school aged children do appear to record higher C_q_ values than adults.

Our study adds that this effect disappears in 12–18-year-olds with suspected Delta variant. This may be confounded by most adults being vaccinated during this period, which could bring C_q_ values up towards those of younger unvaccinated individuals. However, our study cannot prove this. The lower viral loads with wild-type and Alpha SARS-CoV-2 infections suggest that children may be less likely to transmit the virus, however, children may have more prolonged and closer mixing exposures, so this association should be interpreted with caution. Care should be taken when using C_q_ values to determine risk of transmission as this also depends on factors such as duration of viral shedding, length of contact, and possibly symptom status. A study published in 2021 assessing National Health Service (NHS) Test and Trace data suggests that fewer contacts of young people are infected, although the precise role of children in virus transmission is unclear and warrants further investigation [8]. It may be that the Delta variant has removed some of the differences in secondary school aged children.

There are various potential explanations for higher C_q_ values in younger people. It may be that children experience mild disease, clear the virus quicker and therefore have lower average viral loads. Symptom status, swabbing technique and study period did not explain the age effect observed for suspected wild-type or alpha cases (Supplementary Figures 4–6 and Tables S3 and S4).

Another notable finding in our study is that positive tests with suspected Alpha or Delta variant had lower C_q_ values than positives without, suggesting that individuals infected with these variants may be more contagious. Most noticeably, Delta cases have even lower C_q_ values than Alpha cases which relates to higher viral loads and suggests greater transmission risk. An ONS study reported that the Alpha variant (denoted by S gene dropout) had similar C_q_ values to SARS-CoV-2 positive qPCR results without S-gene dropout between 28 September and 2 January [21]. They noted initial differences showing lower C_q_, but this levelled off over time, possibly reflecting a greater proportion of tests being from earlier phases of infection in that phase of the epidemic curve, rather than the Alpha variant causing a higher viral loads per se. The ONS study used random sampling, so it may be that some of the later positive cases identified were post-infectious with low viral load, making it difficult to estimate differences in peak viral load. In contrast, the data from our cohort was collected when individuals chose to attend testing centres, perhaps reflecting an earlier point in their infection cycle. However, our study does suggest higher viral loads for both the Alpha and Delta variant (Figure 4). We do not have genetic sequencing information to confirm the variant type and are only able to infer this from target-gene dropouts vs the date of the test.

A limitation of our study is that C_q_ values have been analysed in a cross-sectional manner, but they will change over the course of an individual’s infection. It is possible that some groups of individuals present for testing at earlier or later stages of their infection and so the changes in C_q_ value reflect more the willingness of groups of individuals to seek testing rather than any real difference in viral load between groups. We do not have any data on onset of symptoms that might allow assessment of where in a viral trajectory an individual was at the point of testing. C_q_ values have been shown to take longer to increase (reduce viral load) in Delta cases than wild-type or Alpha SARS-COV-2 from the date on symptom onset [14]. In our setting differences in C_q_ value may be influenced by differences in test seeking behaviour. However, we are only aware of very limited data on test seeking behaviour at different time periods during the pandemic. Initially, males were less likely to seek testing, as were people living in more deprived areas, and those of non-white ethnicity [17]. However, we do not have systematic information on how this may have changed for later variants. Further, immunity (whether acquired through previous infection or induced by vaccination) is likely to have had an effect on symptom onset, with immune naïve individuals likely to develop symptoms later than vaccinated or previously infected individuals. But there are insufficient data to assess how much this affected the test seeking behaviour and hence C_q_ value.

Median C_q_ values can change depending on the stage of a pandemic and on which groups are testing positive for SARS-CoV-2 [26]. However, the age effects, and variant effects still show in a number of time periods and stages of vaccination coverage (Supplementary Figures S6 and S7).

Our study only considers community testing and so will exclude hospitalised individuals. This may cause a small bias in our analysis if individuals in hospital had more severe symptoms and higher viral loads [27]. However, we believe this is unlikely to change the observation that children have higher C_q_ values than adults, as most children and all but the most elderly were less likely to be admitted to hospital with COVID-19 [26].

We are unable to distinguish between asymptomatic individuals and those who do not have symptoms at the point of test but later go on to develop symptoms. The definition of symptoms is also limited by being self-reported, and hence possibly subject to recall bias, and confusion over what constitutes COVID-19 symptoms. Those with wider (WHO definition) symptoms were encouraged to present for qPCR testing in Liverpool and may have used the more convenient asymptomatic testing sites (rather than for example requesting a home testing kit) but not reported symptoms. In retrospect, the list of symptoms qualifying for qPCR testing could have been expanded to catch more cases of the Delta variant [28].

We do not have access to sequencing data, which means we are relying on the presence/absence of S gene in a positive test to distinguish likely cases of Alpha, Delta and wild-type SARS-COV-2. Further, we rely on a date of 1 April 2021 to distinguish between wild-type and Delta (both of which are usually positive for S gene, N gene and ORF1ab). This may lead to some misclassification of variant type. However, we believe this is likely to be minimal, affecting only the transitions between major variant periods.

Understanding the association between features such as age, and the viral load of an individual is important in targeting COVID-19 responses for maximum control of SARS-CoV-2 transmission. This study provides a reference point for such studies. A major COVID-19 response in the UK has been the use of lateral flow devices to quickly identify and isolate the most infectious individuals [8,15,29]. If children have lower viral loads, on average, than adults, they may shed less antigen, possibly below the limits of detection by lateral flow devices [16]. This concern may have been reduced when the Delta variant was dominant and the viral loads of 12–18-year-olds appear to be broadly comparable to most of the adult population. If lower viral load in children is linked to lower shedding and transmission, this may not be a significant problem. However, further work should assess the link between age, viral load and transmission to inform testing strategies. Conversely, if the Delta variant generally has 2–3 units lower C_q_s than wild-type SARS-CoV-2, this adds value to the use of LFTs, since they are able to detect with reasonable accuracy individuals with low C_q_ [16].

### Conclusion

This study points to age and dominant variant as two important parameters to consider in COVID-19 control strategies, as they are associated with the observed viral load of positive individuals. Further research is needed to understand how vaccination changed viral load, viral kinetics, and transmission potential of SARS-CoV-2 in different age groups. Future pandemic responses might be improved by building this epidemiological surveillance into test and trace strategies.

## Supplementary Data

1448000 Supplementary Material
