# Supplementary material for: Association of SARS-CoV-2 viral load distributions with individual demographics and suspected variant type: results from the Liverpool community testing pilot, England, 6 November 2020 to 8 September 2021
Source: Euro Surveill. 2023 Jan 26;28(4):2200129. doi: 10.2807/1560-7917.ES.2023.28.4.2200129 (PMC9881177; doi:10.2807/1560-7917.ES.2023.28.4.2200129)
Supplement: Supplementary Material [file 2200129_SupplementaryMaterial.pdf]

Supplement to: Association of SARS-CoV-2 viral load distributions with individual demographics and suspected variant type: Results from the Liverpool Community Testing Pilot

This supplementary material is hosted by *Eurosurveillance* as supporting information alongside the article Association of SARS-CoV-2 viral load distributions with individual demographics and suspected variant type: Results from the Liverpool Community Testing Pilot, on behalf of the authors, who remain responsible for the accuracy and appropriateness of the content. The same standards for ethics, copyright, attributions and permissions as for the article apply. Supplements are not edited by *Eurosurveillance* and the journal is not responsible for the maintenance of any links or email addresses provided therein.

This supplementary material reports the mean cycle thresholds and age (Figure S1), the distributions of cycle thresholds by age (Figure S2), the differences between cycle thresholds by age, symptom status and suspected variant type (Figure S3), by age, administration method and suspected variant type (Figure S4), by age and study period (Figure S5) and the counts of number of individuals with a positive PCR related to Supplementary Figures S3 and S4 in Supplementary Tables S1 and S2 respectively.

Additional study details

In the Cheshire and Merseyside, UK region, 6365 positive tests on 6241 individuals were obtained at laboratories other than Lighthouse laboratories. Since these labs did not test for all three of the genes of interest in this study, we have not considered these positives in any of the analysis in this paper.

Pillar 2 testing was available to over 5's. 4208 individuals had age was recorded as 4 or younger, and additionally 13 individuals with age recorded as being over 112. These 4221 individuals were removed from the density plots for Cq by age, and from the regression models and assumed to be data entry errors. Similarly, 220 individuals did not have their sex recorded, and were omitted from the density plots for sex and the regression models.

Is the age effect explained by symptom status, swabbing method or time period?

Supplementary Figure S3 shows that higher Cq values for school age children can be seen in both symptomatic and asymptomatic individuals (except for suspected cases of Delta variant) but is more pronounced in asymptomatic individuals. The numbers of individuals in each category is reported in Supplementary Table S1.

It could be that swabbing technique in young children provides a sample of lower quality due to the difficulties of performing the test. This could be exaggerated in self-administered tests. However, Supplementary Figure S4 shows the Cq distributions by age for self-administered tests and for those done by health care professionals, and in both cases the shifted distributions for 5–11- and 12–18-year-olds remains for both wild-type and alpha variants, and the 5-11-year-old distribution for suspected Delta variant cases. The shift is more pronounced for the health care professionals, possibly suggesting better swabbing quality. Supplementary Table S2 reports the numbers of individuals in each category related to Supplementary Figure S4.

It is known that viral load is associated with increased disease severity. Supplementary Figure S5 shows that the age effect is reduced in later time periods, corresponding to the point at

which lateral flow testing was government policy for secondary school children. It could be that the introduction of twice weekly lateral flow testing has encouraged earlier uptake of PCR testing which is reflected in lower Cq values, recorded earlier in an individual's infection cycle.

Cq differences by Sex

Median regression showed that males had slightly lower Cq values than females (Table 3). However, visual inspection of Figure 2 shows that there is very little difference between males and females in terms of their viral loads, and similar proportions of positive tests were in each of the viral load categories (Table 2). This suggests the statistical significance was largely a result of sample size.

Cq differences by Test Location

Median regression suggested tests taken at home had slightly lower Cq values than tests taken elsewhere (Table 3), although visual inspection of Figure 2 suggests minimal differences.

Cq differences by Administration Method

There were a large number of individuals for whom it was not known how the PCR test was administered. Multivariable quantile regression shows statistically significant differences in median Cq of self-administered tests compared to those administered by health care professionals, although the effect size is small (Table 3 and Figure 2).

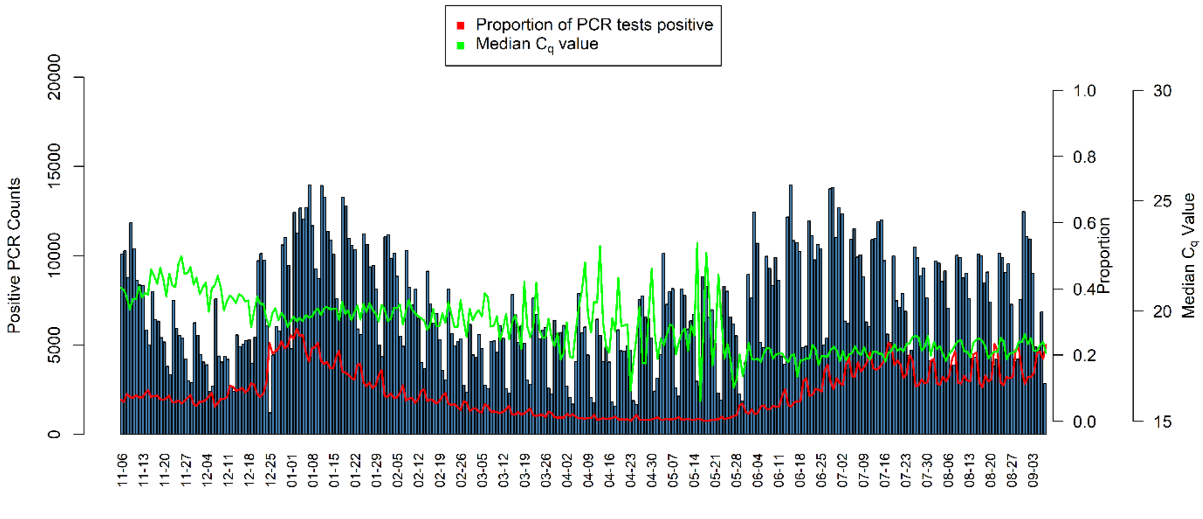

Figure S1: Number of positive SARS-CoV-2 infected individuals identified by testing over the study period. The median cycle threshold (Cq) for the mean of all genes present is shown in green. The proportion of positive cases out of all PCR tests in a day is shown in red.

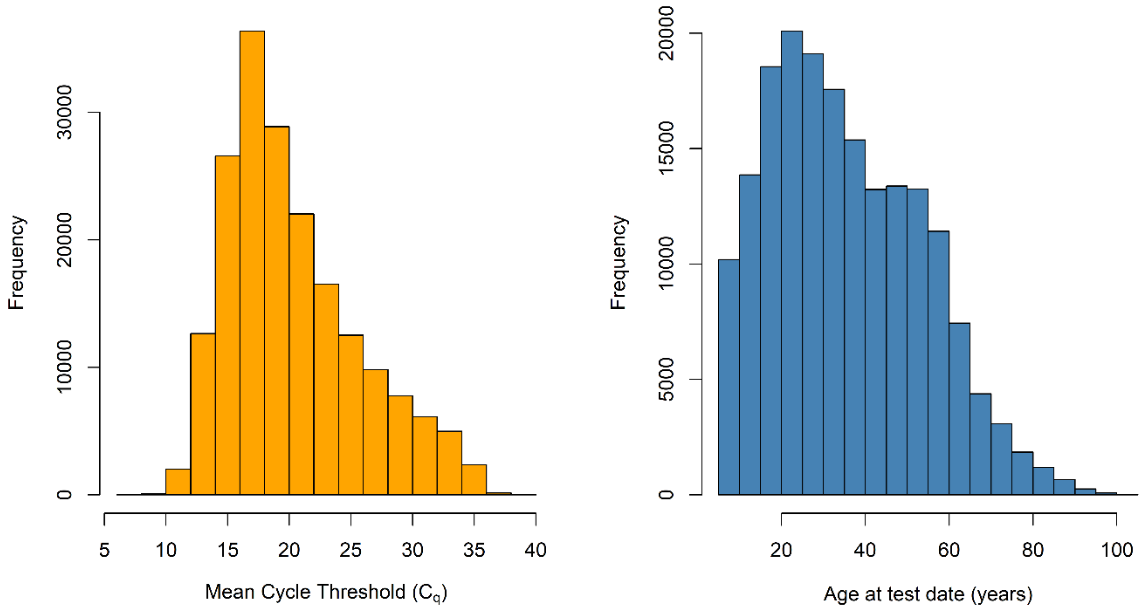

Figure S2: Distributions of cycle threshold, and Age within the study cohort.

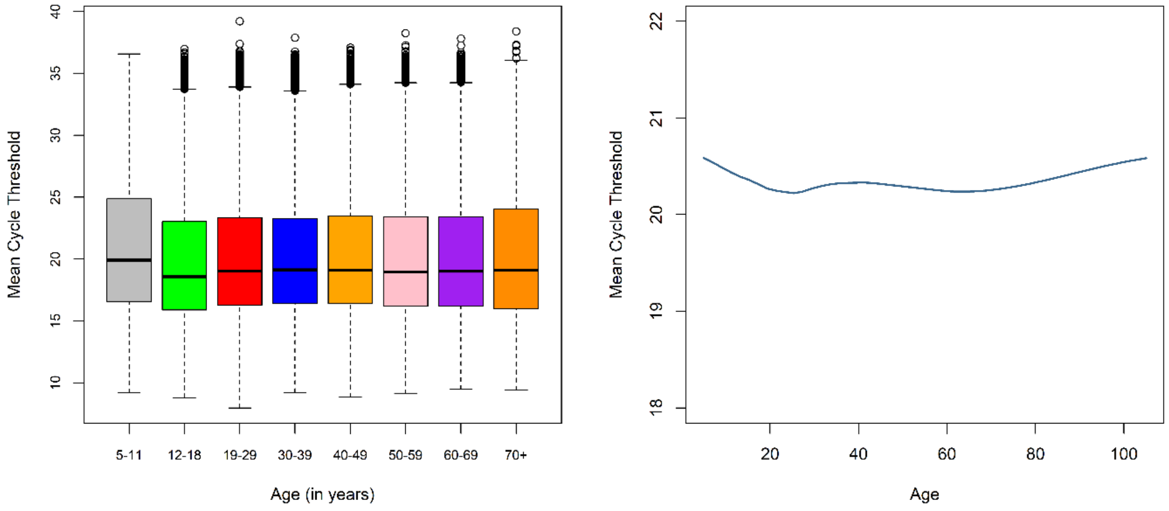

Figure S3: Distribution of mean PCR cycle threshold (Cq), by age categories (left panel) and a LOESS smoothed plot showing the non-linear relationship between Cq against age (right panel). Ages recorded on all positive PCR results with Cq values between 6th November 2020 and 9th June 2021 are considered.

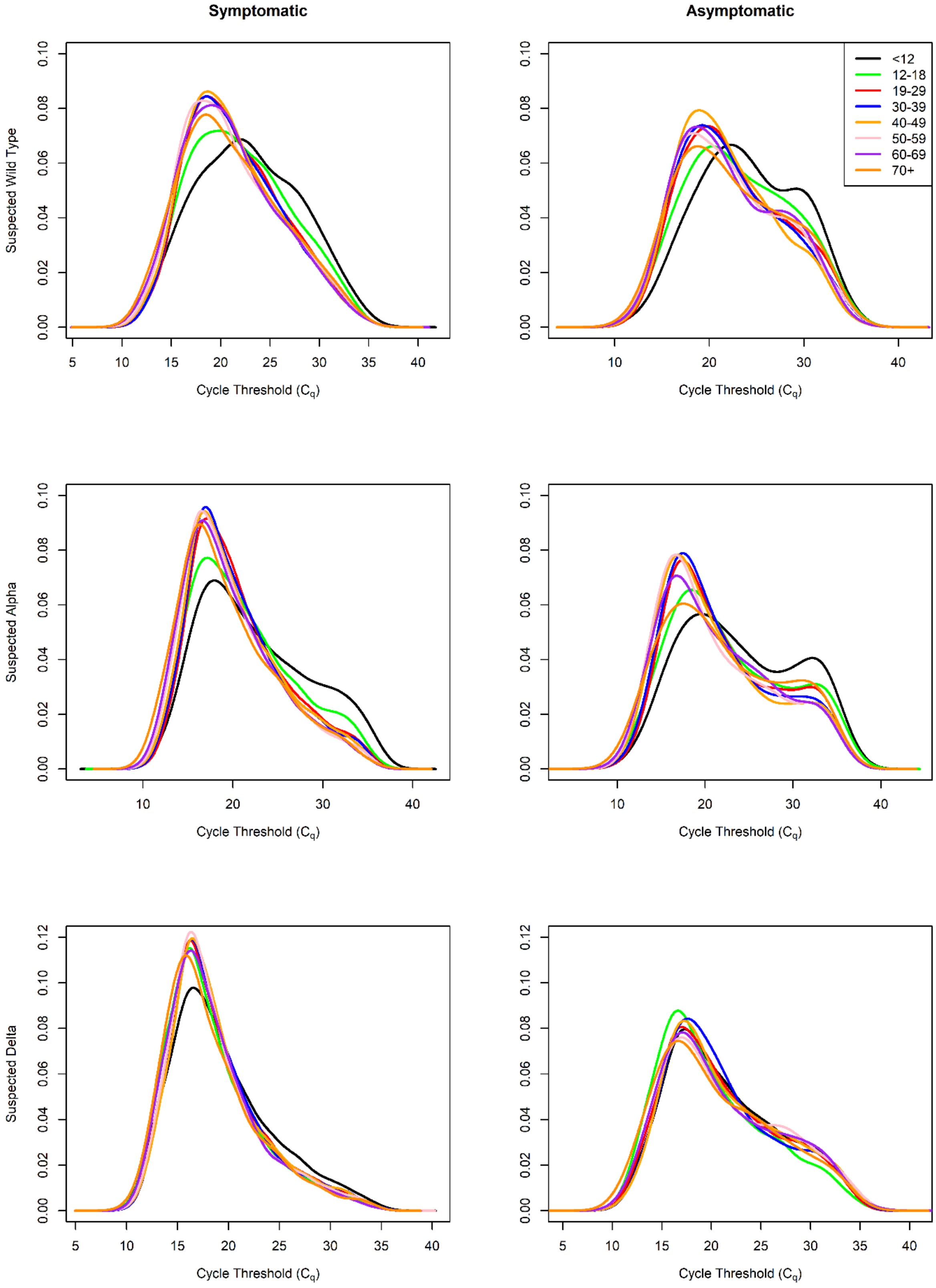

Figure S4: Comparison of RT-qPCR cycle thresholds (Cq) by age, symptom status and suspected variant.

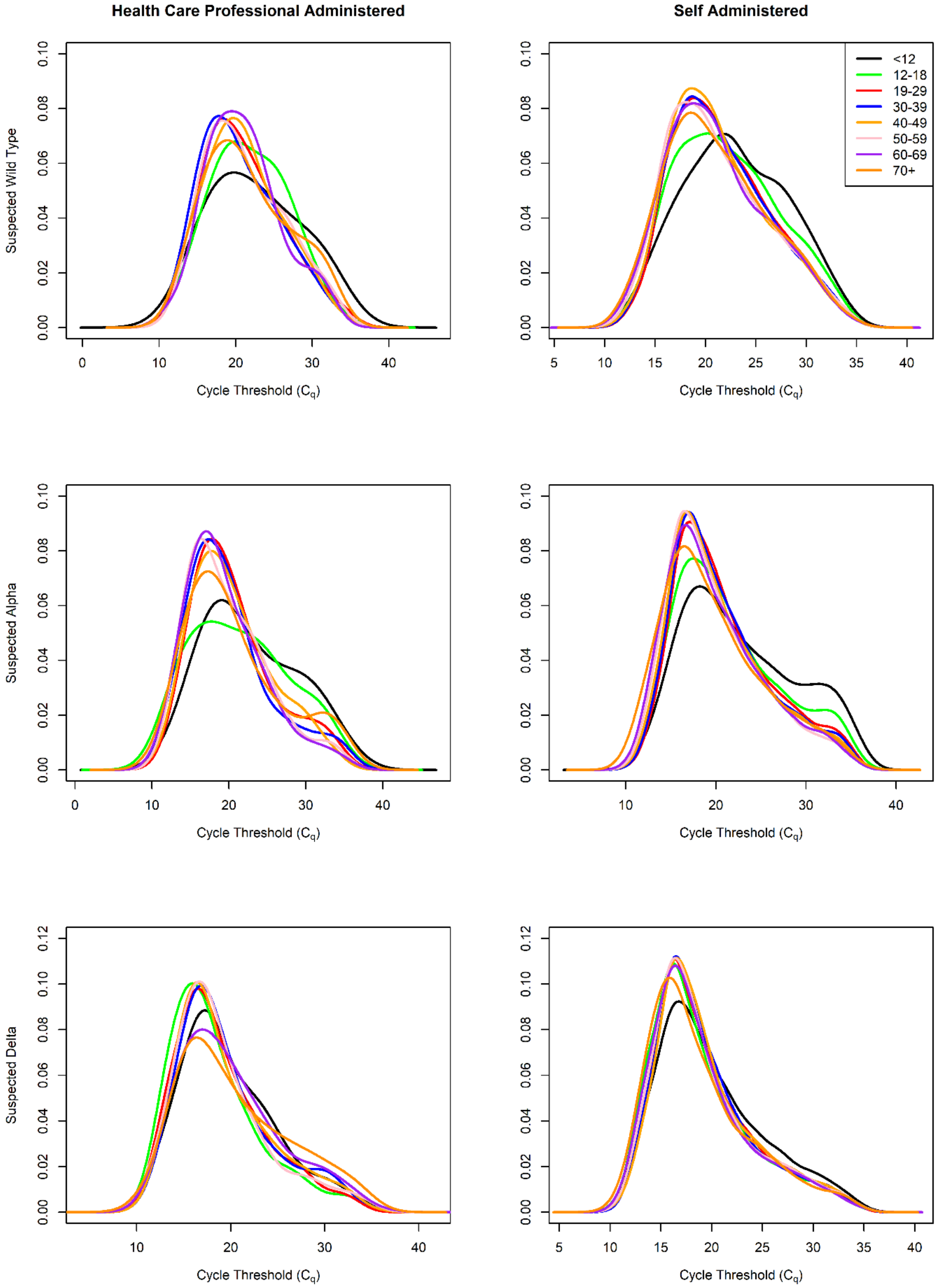

Figure S5: Comparison of RT-qPCR cycle thresholds (Cq) by age, administration method and suspected variant.

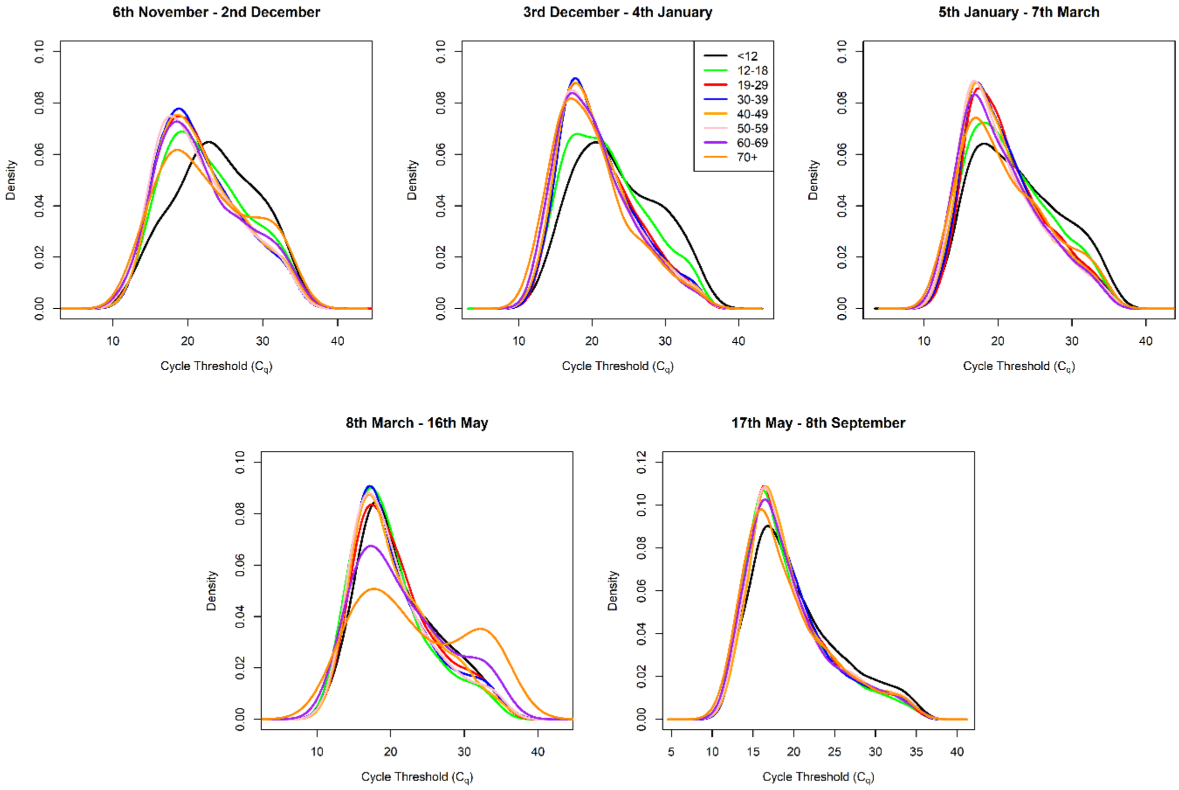

Figure S6: Comparison of RT-qPCR cycle thresholds (Cq) by age and time epoch during the COVID-19 pandemic.

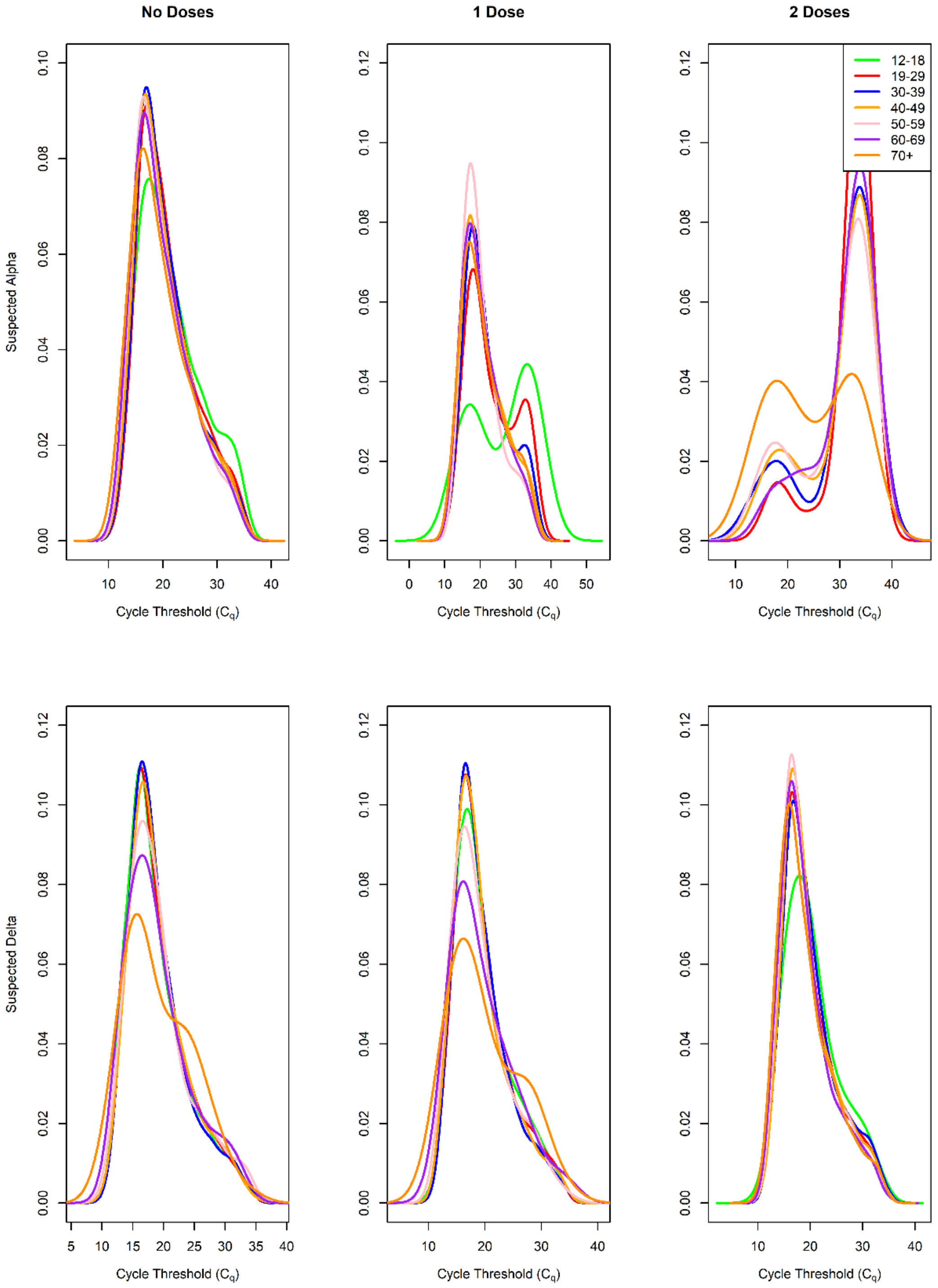

Figure S7 : Comparison of RT-qPCR cycle thresholds (Cq) by age, variant type and number of vaccine doses.

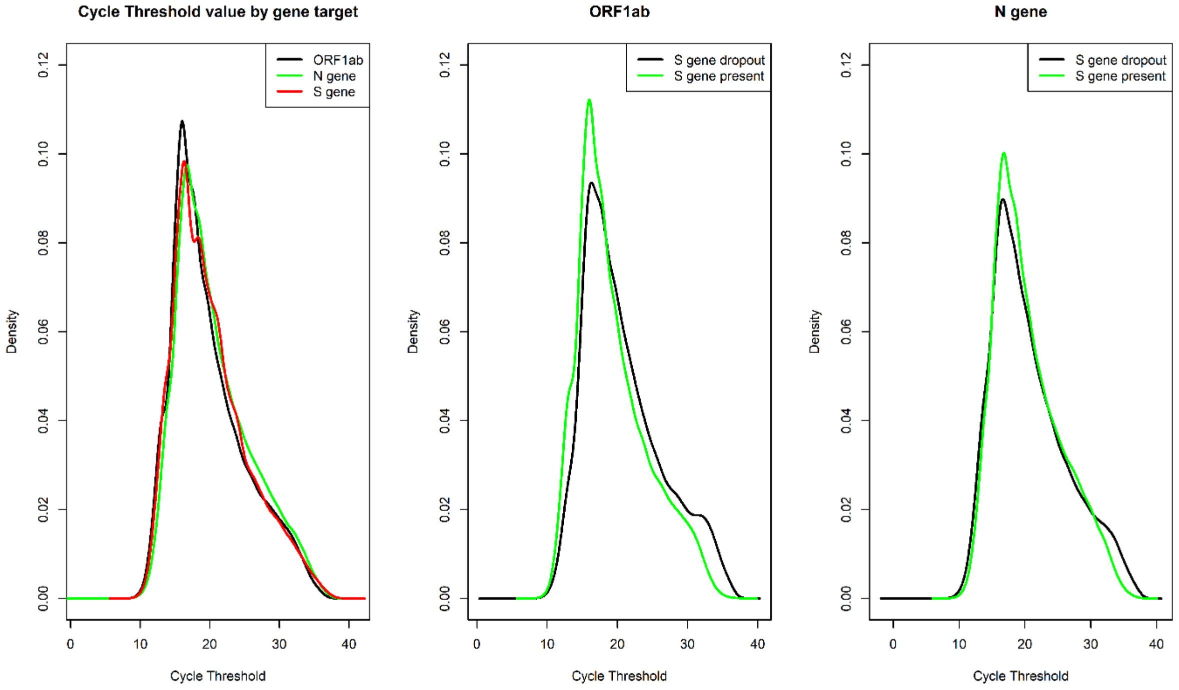

Figure S8: Comparison of RT-qPCR cycle thresholds (Cq) by gene target and presence or absence of S-gene.

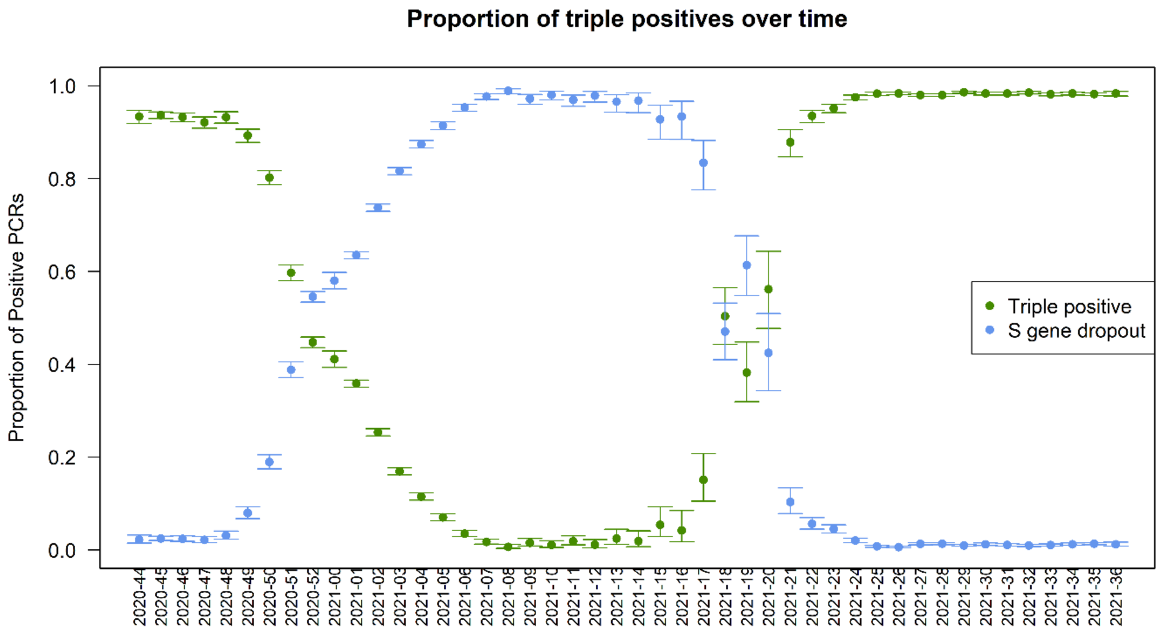

Figure S9: Proportion of positive PCRs with all three target genes positive (S-gene, N-gene and ORF1ab) and of those with S-gene drop out (SGTF) by week of pandemic.

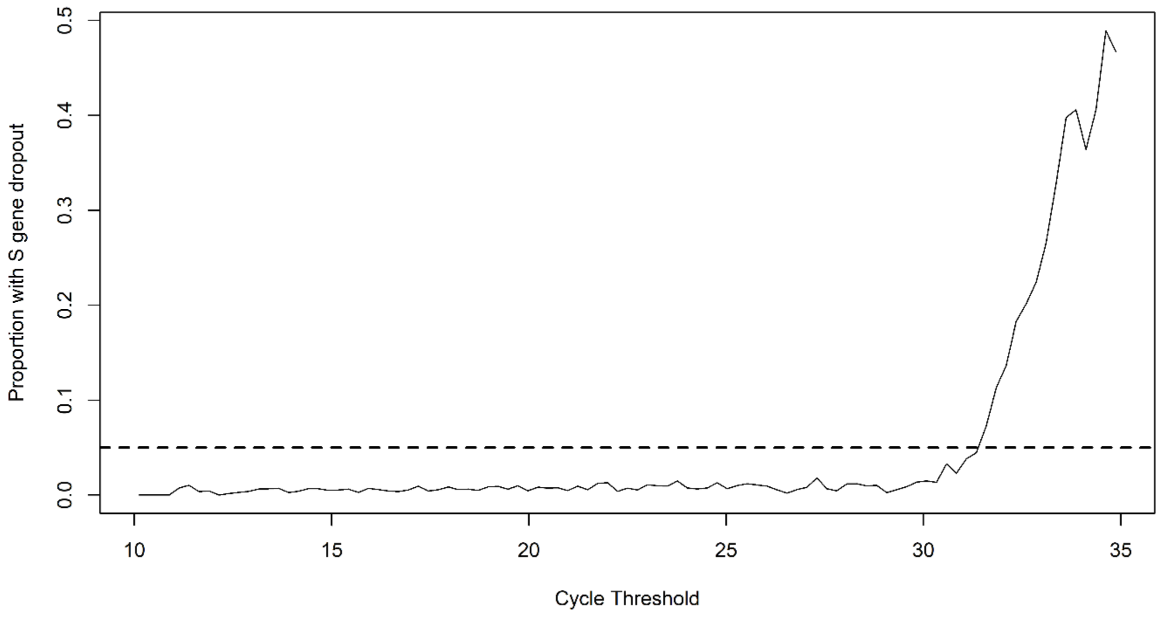

Figure S10: Proportion of positive PCRs with S-gene dropout by mean cycle threshold value (calculated using all present gene targets). Only samples taken later than 1st May 2021 (the Delta period) were included in this figure.

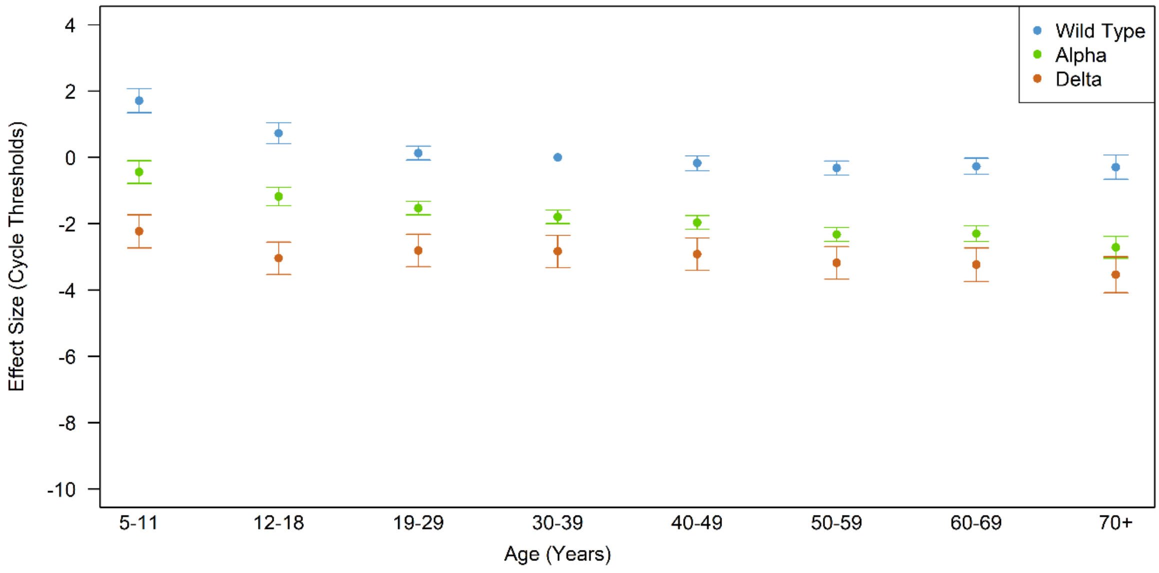

Figure S11: Association between median cycle threshold, and age, variant interactions. Contrasts are relative to a 30–39-year-old with wild-type COVID-19. This analysis was restricted to samples with cycle threshold less than 31.

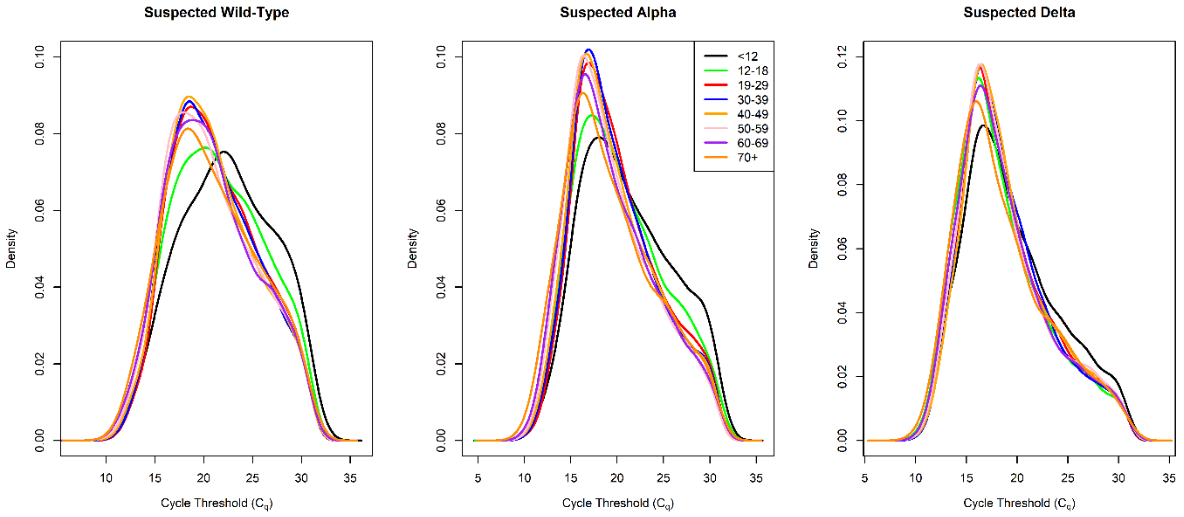

Figure S12: Distribution of RT-qPCR cycle threshold (Cq) values by age and suspected variant type. This analysis was restricted to samples with cycle threshold less than 31.

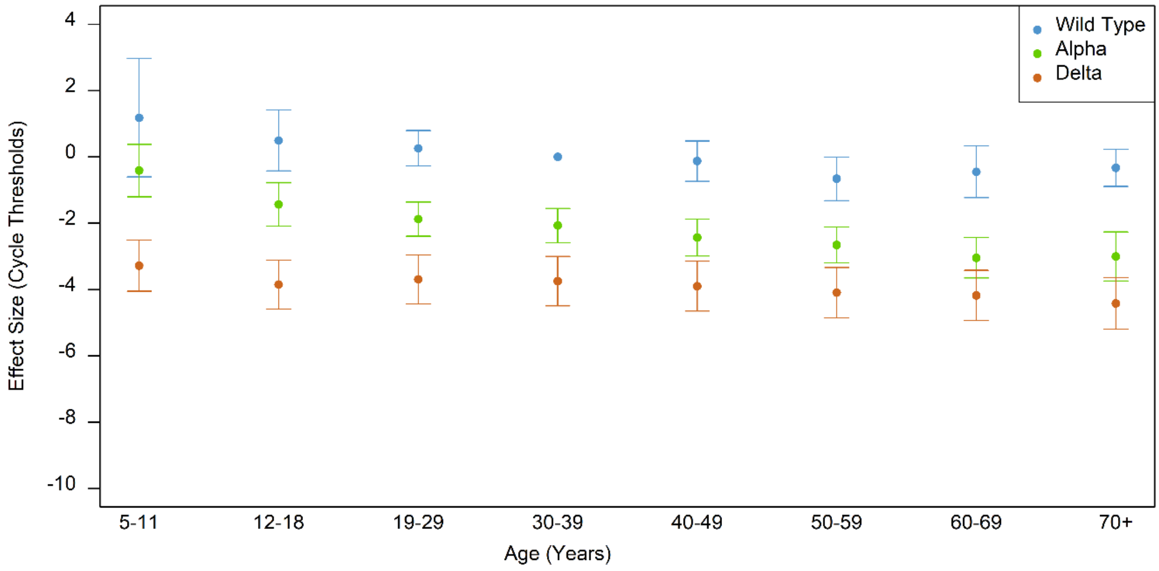

Figure S13: Association between median cycle threshold, and age, variant interactions. Contrasts are relative to a 30-39 year old with wild-type COVID-19. This analysis was restricted to individuals with a positive LFT in the 14 days prior to the positive PCR.

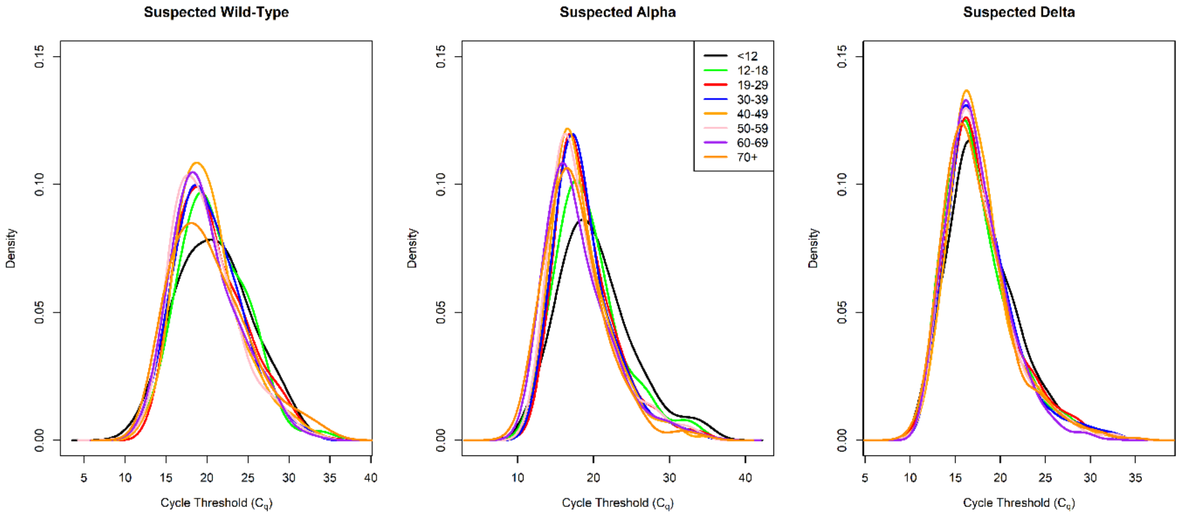

Figure S14: Distribution of RT-qPCR cycle threshold (Cq) values by age and suspected variant type. This analysis was restricted to individuals with a positive LFT in the 14 days prior to the positive PCR.

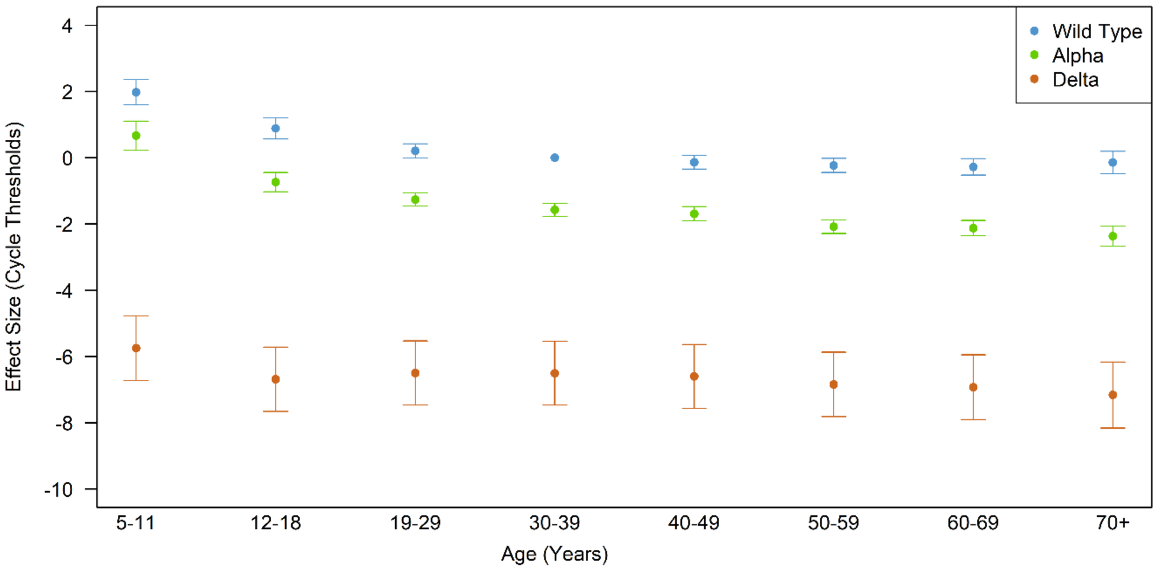

Figure S15: Association between median cycle threshold, and age, variant interactions. Contrasts are relative to a 30-39 year old with wild-type COVID-19. This analysis considered the positive PCR with the lowest cycle threshold (rather than the first) for each individual.

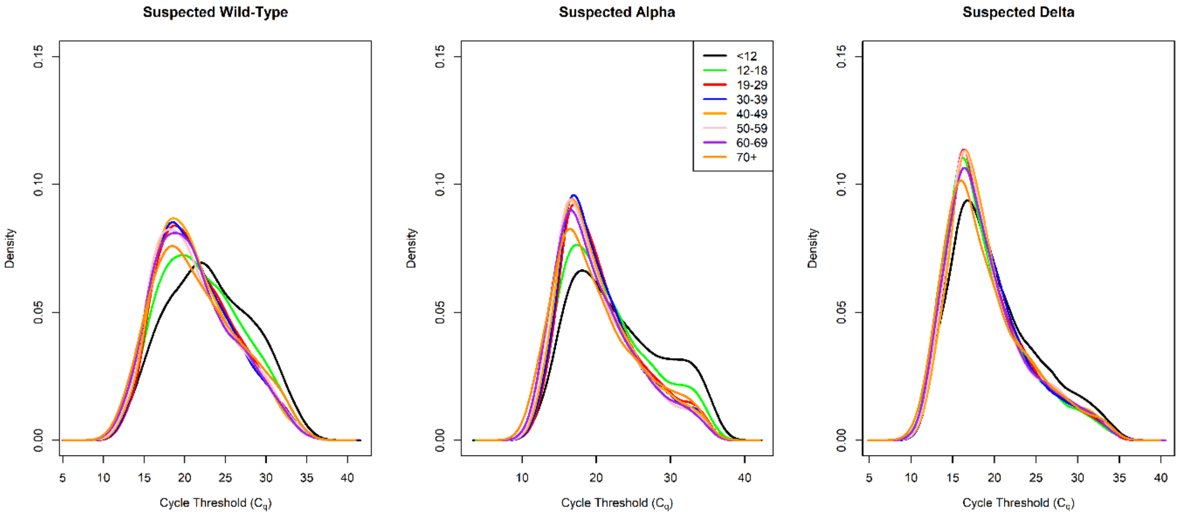

Figure S16: Distribution of RT-qPCR cycle threshold (Cq) values by age and suspected variant type. This analysis considered the positive PCR with the lowest cycle threshold (rather than the first) for each individual.

Table S1: PCR Cq values for different demographic groups, with proportions corresponding to low, medium, high and very high viral loads. The median column reports the median, and interquartile range of the mean of any available Cq gene values.

	**Individual Characteristics**	**Median PCR Cycle Threshold (Cq)**	**Cq <18.3 No. (%)**	**Cq 18.3-24.4 No. (%)**	**Cq 24.4-30.5 No. (%)**	**Cq 30.5-35 No. (%)**
Approximate Viral Load (RNA copies/ml)			$>10^6$	$>10^4$-10^6	$>10^2 – 10^4$	$\leq 10^2$
	Total	19.1 (16.3, 23.5)	82757 (44)	65274 (35)	28865 (15)	11925 (6)
Age	5-11	19.8 (16.5, 24.7)	4633 (40)	4005 (34)	2061 (18)	982 (8)
	12-18	18.6 (15.9, 23.0)	10669 (48)	7284 (33)	3170 (14)	1260 (6)
	19-29	19.0 (16.3, 23.3)	18873 (44)	15098 (35)	6384 (15)	2447 (6)
	30-39	19.1 (16.4, 23.3)	14638 (43)	12168 (36)	4971 (15)	2011 (6)
	40-49	19.1 (16.4, 23.5)	11590 (43)	9375 (35)	4093 (15)	1623 (6)
	50-59	19.0 (16.2, 23.4)	11394 (45)	8634 (34)	3923 (15)	1528 (6)
	60-69	19.0 (16.2, 23.4)	5828 (44)	4513 (34)	2022 (15)	778 (6)
	70+	19.1 (16.0, 24.0)	3471 (44)	2513 (32)	1233 (16)	619 (8)
Sex	Male	18.9 (16.1, 23.2)	41322 (46)	30602 (34)	13236 (15)	5300 (6)
	Female	19.3 (16.4, 23.8)	41351 (42)	34618 (35)	15611 (16)	6612 (7)
Ethnicity	White	19.0 (16.2, 23.4)	76523 (44)	59622 (34)	26067 (15)	10652 (6)
	Black	20.7 (17.5, 26.0)	563 (33)	638 (37)	337 (20)	170 (10)
	Asian	19.5 (16.6, 24.2)	1666 (41)	1397 (35)	687 (17)	285 (7)
	Mixed or multiple ethnic groups	19.5 (16.6, 23.8)	1111 (40)	1036 (37)	438 (16)	184 (7)
	Another ethnic group	19.8 (16.6, 24.7)	508 (38)	472 (35)	243 (18)	108 (8)
	Prefer not to say	19.8 (16.6, 24.8)	2386 (39)	2109 (34)	1093 (18)	526 (9)
Symptoms Reported	Yes	18.8 (16.1, 22.9)	68031 (46)	51658 (35)	20858 (14)	7052 (5)
	No	20.5 (16.9, 26.2)	14726 (36)	13616 (33)	8007 (19)	4873 (12)
Home Test	Yes	19.0 (16.0, 23.8)	16978 (45)	12305 (32)	5770 (15)	2835 (7)
	No	19.1 (16.3, 23.5)	65779 (44)	52969 (35)	23095 (15)	9090 (6)
Administration Method	Healthcare professional	18.9 (16.1, 23.3)	3773 (45)	2852 (34)	1214 (15)	524 (6)
	Self-administered	19.0 (16.3, 23.4)	65874 (44)	52033 (35)	22458 (15)	8741 (6)
	Not known	19.5 (16.4, 24.4)	13110 (42)	10389 (33)	5193 (17)	2660 (8)
Test period	6th November – 2nd December	21.1 (17.7, 25.9)	3488 (30)	4447 (38)	2525 (22)	1136 (10)
	3rd December – 4th January	20.0 (16.9, 24.2)	8720 (37)	9224 (39)	4211 (18)	1464 (6)
	5th January – 7th March	20.0 (16.8, 24.5)	19976 (38)	19452 (37)	9671 (18)	3795 (7)
	8th March – 16th May	19.2 (16.4, 24.0)	1786 (42)	1436 (34)	640 (15)	344 (8)
	17th May – 8th September	18.2 (15.9, 22.3)	48787 (2)	30715 (32)	11818 (12)	5186 (5)
Variant	Unknown	33.6 (32.1, 34.6)	66 (3)	105 (5)	122 (6)	1652 (85)
	Wild Type	20.9 (17.8, 25.1)	9819 (29)	14165 (42)	7593 (23)	1947 (6)
	Suspected Alpha	19.5 (16.5, 24.1)	24086 (41)	20361 (35)	9421 (16)	4623 (8)
	Suspected Delta	18.2 (15.8, 22.0)	48786 (51)	30643 (32)	11729 (12)	3703 (4)
Vaccine Doses	0	19.4 (16.4, 23.8)	57087 (42)	48297 (35)	22129 (16)	8746 (6)
	1	18.5 (16.1, 22.7)	10052 (48)	6885 (33)	2728 (13)	1209 (6)
	2	18.4 (16.0, 22.6)	15618 (49)	10092 (32)	4008 (13)	1970 (6)

Table S2: Age and suspected VOC specific contrast in PCR cycle threshold based on the multivariate quantile regression model. The comparison for suspected wild-type is presented in Table 2.

Suspected VOC	Age (vs 30-39)	Contrast	95% CI	p-value
Alpha	5-11	2.20	(1.81, 2.58)	<0.001
	12-18	0.81	(0.55, 1.07)	<0.001
	19-29	0.31	(0.15, 0.48)	<0.001
	40-49	-0.13	(-0.32, 0.06)	0.176
	50-59	-0.55	(-0.72, -0.37)	<0.001
	60-69	-0.56	(-0.78, -0.35)	<0.001
	70+	-0.79	(-1.08, -0.5)	<0.001
Delta	5-11	0.75	(0.58, 0.91)	<0.001
	12-18	-0.18	(-0.3, -0.06)	0.004
	19-29	0.06	(-0.05, 0.17)	0.277
	40-49	-0.12	(-0.25, 0.01)	0.075
	50-59	-0.37	(-0.52, -0.21)	<0.001
	60-69	-0.47	(-0.63, -0.3)	<0.001
	70+	-0.69	(-0.92, -0.45)	<0.001

Table S3: Counts of the number of individuals with positive PCR tests by age, suspected variant type and symptom status.

	Suspected Wild-Type		Suspected Alpha		Suspected Delta	
Age	Asymptomatic	Symptomatic	Asymptomatic	Symptomatic	Asymptomatic	Symptomatic
5-11	257	1002	712	1945	2848	5881
12-18	431	2145	812	3159	4235	11407
19-29	1119	5483	2250	9574	5007	19022
30-39	962	5262	1911	9195	3446	12711
40-49	779	4452	1624	7583	2794	9139
50-59	999	4973	1800	8452	2188	6795
60-69	605	2564	1037	4221	1211	3353
70+	490	1434	844	1977	965	1965

Table S4: Counts of the number of individuals with positive PCR tests by age, suspected variant type and administration method.

	Suspected Wild-Type		Suspected Alpha		Suspected Delta	
Age	Health Care Professional	Self	Health Care Professional	Self	Health Care Professional	Self
5-11	19	1014	51	2158	242	6983
12-18	167	2069	156	3148	589	12387
19-29	338	5525	461	9762	908	19400
30-39	266	5204	388	9199	637	13060
40-49	295	4268	378	7436	487	9572
50-59	404	4677	552	7972	395	6871
60-69	237	2307	296	3841	250	3316
70+	133	1152	171	1524	368	1824

Table S5: Quantile Regression Model parameters for sensitivity analysis

		Minimum Cq per individual			Confirmatory PCR			Cq < 31		
		Parameter	95% CI	p-value	Parameter	95% CI	p-value	Parameter	95% CI	p-value
Sex (reference = female)	Male	-0.37	(-0.41, -0.32)	<0.001	-0.27	(-0.37, -0.18)	<0.001	-0.35	(-0.39, -0.3)	<0.001
Symptoms (reference = No)	Yes	-1.51	(-1.58, -1.44)	<0.001	1.27	(0.54, 1.8)	0.002	-1.24	(-1.31, -1.17)	<0.001
Ethnicity (reference = White)	Black	1.33	(1.02, 1.65)	<0.001	0.31	(-0.06, 0.77)	0.237	1.14	(0.81, 1.44)	<0.001
	Asian	0.12	(-0.06, 0.29)	0.221	0.99	(0.12, 1.72)	0.038	0.15	(-0.01, 0.33)	0.14
	Another ethnic group	0.47	(0.23, 0.67)	0.001	0.9	(0.56, 1.22)	<0.001	0.51	(0.25, 0.78)	0.001
	Mixed or multiple ethnic groups	0.58	(0.37, 0.77)	<0.001	0.42	(0.11, 0.75)	0.013	0.58	(0.41, 0.8)	<0.001
	Prefer not to say	0.43	(0.3, 0.55)	<0.001	0.4	(0.26, 0.51)	<0.001	0.38	(0.24, 0.54)	<0.001
Home Test (reference = No)	Yes	-0.18	(-0.25, -0.11)	<0.001	0.87	(-0.02, 1.24)	0.002	-0.2	(-0.27, -0.13)	<0.001
Test Period (reference = 6th Nov – 2nd Dec)	3rd Dec – 4th Jan	-0.13	(-0.26, -0.01)	0.048	1.66	(0.75, 2.08)	<0.001	0.06	(-0.05, 0.18)	0.389
	5th Jan – 7th Mar	0.46	(0.33, 0.59)	<0.001	2.08	(1.26, 2.81)	<0.001	0.69	(0.55, 0.81)	<0.001
	8th Mar – 16th May	0.35	(0.1, 0.57)	0.019	0.21	(-0.04, 0.52)	0.171	0.21	(-0.01, 0.45)	0.127
	17th May – 8th Sep	3.84	(3.33, 4.59)	<0.001	1.25	(0.47, 1.7)	<0.001	0.36	(-0.03, 0.83)	0.143
Administration Method (reference = Health Care Professional	Unknown	0.23	(0.11, 0.36)	0.003	0.19	(-0.03, 0.44)	0.155	0.26	(0.13, 0.38)	0.001
	Self administered	0.19	(0.09, 0.31)	0.006	0.28	(0.14, 0.4)	0.001	0.24	(0.11, 0.34)	0.001
LFT tests in previous 14 days prior to positive LFT (reference = 0)	1	-1.02	(-1.08, -0.96)	<0.001	0.46	(0.29, 0.62)	<0.001	-0.86	(-0.92, -0.8)	<0.001
	2+	-0.99	(-1.06, -0.9)	<0.001	0.14	(-1.13, 0.81)	0.791	-0.81	(-0.9, -0.74)	<0.001
Vaccine Doses (reference =0)	1	0.34	(0.25, 0.41)	<0.001	-0.06	(-0.52, 0.42)	0.841	0.28	(0.2, 0.35)	<0.001
	2	0.53	(0.44, 0.61)	<0.001	-0.24	(-0.87, 0.24)	0.51	0.44	(0.37, 0.53)	<0.001
